# Genetic Diversity and Phylogenetic Analysis of Human Bocavirus 2 in Pediatric Patients with Acute Gastroenteritis in Taiwan

**DOI:** 10.3390/ijerph17031086

**Published:** 2020-02-08

**Authors:** Marco Yung-Cheng Lin, Hsiu-Chuan Chan, Hsin Chi, Shu-Chun Chiu, Zaiga Nora-Krukle, Santa Rasa-Dzelzkaleja, Anda Vilmane, Modra Murovska, Jih-Hui Lin, Hsin-Fu Liu

**Affiliations:** 1 Department of Medical Research, MacKay Memorial Hospital, Taipei 10449, Taiwan; lin65mt@gmail.com; 2 Department of Nursing, Shu-Zen junior College of Medicine and Management, Kaohsiung 82144, Taiwan; 3 Lipid Science and Aging Research Center, Kaohsiung Medical University, Kaohsiung 80708, Taiwan; earth1981709@hotmail.com; 4 Department of Pediatrics, MacKay Children’s Hospital and MacKay Memorial Hospital, Taipei 10449, Taiwan; chi4531@gmail.com; 5 Department of Medicine, MacKay Medical College, New Taipei 25245, Taiwan; 6 Center of Diagnostics and Vaccine Development, Centers for Disease Control, Taipei 11561, Taiwan; schiu@cdc.gov.tw; 7 Institute of Microbiology and Virology, Riga Stradins University, LV-1067 Riga, Latvia; Zaiga.Nora@rsu.lv (Z.N.-K.); Santa.Rasa@rsu.lv (S.R.-D.); Anda.Vilmane@rsu.lv (A.V.); Modra.Murovska@rsu.lv (M.M.); 8 Department of Bioscience and Biotechnology, National Taiwan Ocean University, Keelung 20224, Taiwan

**Keywords:** human bocavirus 2, acute gastroenteritis, phylogenetic analysis, genotype

## Abstract

Human bocavirus (HBoV) is a causative agent of respiratory and gastrointestinal diseases worldwide. Four HBoV species (HBoV1-4) have been identified so far. Although a previous report has documented the HBoV association with acute gastroenteritis (AGE) in Taiwan, their epidemiology, genetic diversity, and phylogenetic relationships remain unclear. In this study, we focused on an investigation of these unsolved issues, which will help to reveal molecular epidemiology and phylogeny of the circulating HBoV2 in Taiwan. A total of 176 stool samples were collected from children with AGE for this study. PCR amplification and sequencing on the VP1 gene region were used to identify species. Phylogenetic analysis was conducted by maximum-likelihood and neighbor-joining methods. Selection pressure was also estimated to obtain HBoV evolutionary information. Our results showed the prevalence of HBoV in AGE children was 8.5%, of which HBoV1 was the predominant species (6.3%), followed by HBoV2 (2.3%). Phylogenetic analysis showed those Taiwanese HBoV2 strains have significant genetic variability and can be divided into two clusters. One belongs to HBoV2 genotype A and the other forms an independent unclassified cluster. The nucleotide distance between that independent cluster and the known HBoV2 genotypes was more than 5%, suggesting a new HBoV2 genotype. No positive selection site was found and the virus was under purifying selection. This is the first report to reveal HBoV2 genetic diversity and phylogenetic relationships among AGE children in Taiwan. We find that HBoV2 may have been introduced into the country by multiple origins, and a potential new HBoV2 genotype is proposed.

## 1. Introduction

Human bocavirus (HBoV) is small, non-enveloped, single-stranded DNA virus. HBoVs are members of the family *Parvoviridae* and belonged to *Parvovirinae* subfamily, *Bocaparvovirus* genus. Three open reading frames (ORFs) encode two major nonstructure proteins (NS1 and NP1) and two structure proteins (VP1/VP2) were recognized on the HBoVs genome [1].

Currently, HBoVs are classified into four species: HBoV1–4, based on the nucleotide divergence of the VP1 gene region [2]. The first HBoV, named HBoV1, was recognized in 2005 from a child with a respiratory infection [1]. Since 2009, several novel genotypes were identified from fecal specimens and were named HBoV2 to HBoV4. HBoV2 was identified from children with non-polio acute flaccid paralysis (AFP) in Pakistan [3]. HBoV3 was discovered from stool samples of Australian children with diarrhea [4]. The fourth genotype of HBoVs, HBoV4, was identified from children with non-polio AFP from Nigeria and Tunisia [2].

HBoVs cause a variety of clinical symptoms and can be isolated from several types of specimens that include nasopharyngeal aspirates, fecal, blood, and cerebrospinal fluid [4,5,6,7,8,9]. HBoV1 is most commonly detected from respiratory specimens, while HBoV2-4 is often detected in fecal specimens [4,5,6,10]. Furthermore, previous literature has shown that HBoVs are considered to be highly diverse and frequently recombinant pathogens [2,11,12]. Therefore, worldwide surveillance of HBoVs’ genetic evolution is necessary.

Viral gastroenteritis is a common and significant gastrointestinal disease for children worldwide [13]. Several viral pathogens are associated with acute gastroenteritis (AGE) in Taiwan, including rotaviruses, noroviruses, adenoviruses, and astroviruses [14,15]. Although a previous report has documented the HBoV association with AGE in Taiwan [16], their epidemiology, genetic diversity, and phylogenetic relationships remain unclear. It is essential to study these issues. In this study, we focused on an investigation of the prevalence, phylogenetics, and evolution of HBoVs from pediatric patients with AGE, which will help to reveal the molecular epidemiology and phylogeny of the circulating HBoV2 in Taiwan.

## 2. Materials and Methods

### 2.1. Specimen Selection and Ethics Statement

A total of 176 stool samples were collected from the MacKay Memorial Hospital from September 2013 to June 2015. Children with a clinical diagnosis of viral AGE were inducted in this study. Other than age, no selection criteria were applied. The study of ethical approval was obtained from the MacKay Memorial Hospital Institutional Review Board (13MMHIS285). All samples were de-identified and analyzed anonymously.

### 2.2. Virus Detection and Genotype Classification

The viral DNA was extracted from stool suspension samples using the QIAamp DNA Mini Kit (Qiagen, CA), according to the manufacturer’s protocol. Eluted DNA was stored at −70 °C before use. The nested PCR targeting the VP1/2 region for the detection of both HBoV genotypes and its reaction conditions is according to the article by Kapoor et al. [2]. First-round PCR primers were AK-VP-F1: (CGCCGTGGCTCCTGCTCT) and AK-VP-R1: (TGTTCGCCATCACAAAAGATGTG) and second-round primers were AK-VP-F2: (GGCTCCTGCTCTAGGAAATAAAGAG) and AK-VP-R2: (CCTGCTGTTAGGTCGTTGTTGTATGT) [2].

PCR amplification was done by Sensoquest Labcycler (SensoQuest GmbH) with TaKaRa premix Taq DNA polymerase (TaKaRa Bio INC). Positive PCR products were purified with QIAquick PCR purification kits (Qiagen, CA) and subject to direct sequencing by BigDye Terminator v3.1 cycle sequencing kit reagents on an ABI Prism 3730 DNA analyzer (Applied Biosystems). The HBoV sequences were implemented in BLAST on the NCBI website for genotype classification and reference sequences were obtained for later phylogenetic analysis (Table 1).

### 2.3. Phylogenetic Analyses

The dataset of HBoV2 partial VP1 gene sequences (456 base pair) used for phylogenetic analysis were composed of seven sequences from Taiwan, including three sequences from Centers for Disease Control (CDC), Taiwan; four unpublished sequences from Latvia (provided by Augusts Kirhensteins Institute of Microbiology and Virology, Riga Stradins University, Riga, Latvia); and fifty-nine HBoV2 reference sequences from the NCBI database (Table 1), including HBoV2A (GenBank accession numbers (AC): EU082213 and GQ506639) and HBoV2B (AC: FJ973560). The multiple sequence alignment was done using the Muscle method [17] and the estimation of nucleotide distance was done by the p-distance method implemented in MEGA software version 7 (Available online: http://www.megasoftware.net) [18]. The selection of the best-fit nucleotide substitution model was carried out by the smart model selection software implemented in the PhyML web server [19]. The phylogenetic trees were constructed using the maximum-likelihood (ML) method implemented in PhyML 3.0 (Available online: http://www.atgc-montpellier.fr/phyml/) [20] and the neighbor-joining (NJ) method was implemented in MEGA software (Available online: http://www.megasoftware.net). Branch support of the phylogenetic trees was evaluated by bootstrap analysis of 1000 replicates.

### 2.4. Selection Pressure of VP1 Protein Genes

The same data used for phylogenetic analysis was also used to determine the selection pressures on the VP1 protein of HBoV. The ratio of non-synonymous substitutions (dN) and synonymous substitutions (dS) per site based on ML tree under the appropriate substitution model has been estimated using the single likelihood ancestor counting (SLAC), fixed effects likelihood (FEL) methods with a significance level of 0.05 [21]. Bayesian tests for selection acting on individual sites were using FUBAR with posterior probabilities on 0.95 [22]. All methods were implemented in the HyPhy package and accessed through the Datamonkey web-server interface (http://www.datamonkey.org) [23].

## 3. Results

One hundred and seventy-six stool samples were collected from pediatric patients with AGE from September 2013 to June 2015 in MacKay Memorial Hospital, Taiwan. Fifteen stool samples (8.52%) were detected positive for HBoV PCR. The positive PCR products were sequenced and then identified the species using the BLAST service on the NCBI website. Our result showed there were eleven stool samples identified as HBoV1 (6.25%) and four as HBoV2 (2.27%). No HBoV3 and HBoV4 were detected in this study.

### 3.1. Sequence Comparison and Phylogenetic Analysis of HBoV2

**Figure 1:**
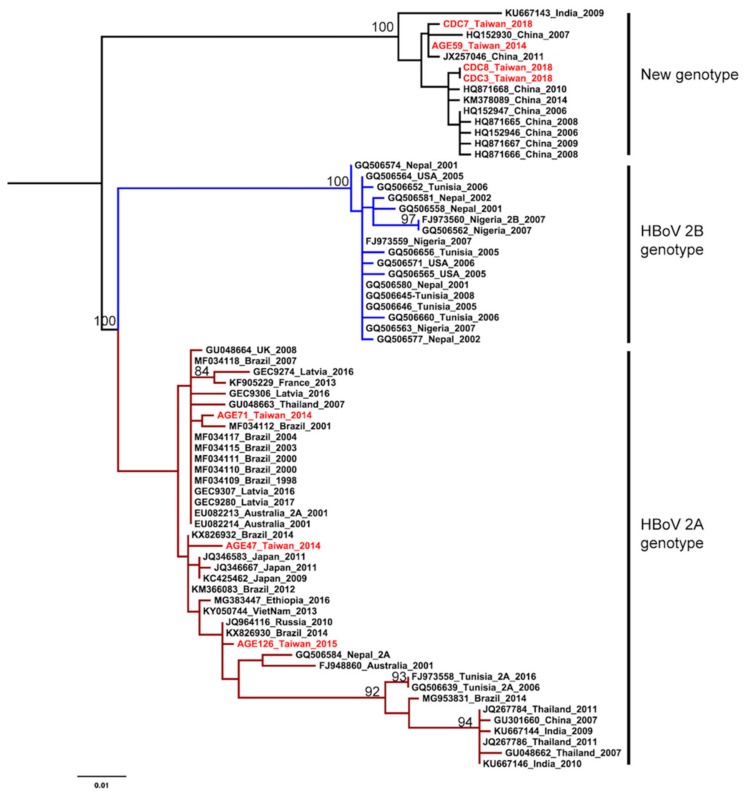
Phylogenetic analysis of human bocavirus 2 using the maximum likelihood method. The phylogenetic tree was reconstructed based on the HBoV2 partial VP1 gene. The stability of the tree topology was evaluated by using 1000 bootstrap replicates. Only bootstrap values greater than 75% are shown on the branch. The red texts represent HBoV2 isolates in Taiwan from the children with AGE. Brown and blue clades are represented HBoV2A and HBoV2B, respectively.

**Figure 2:**
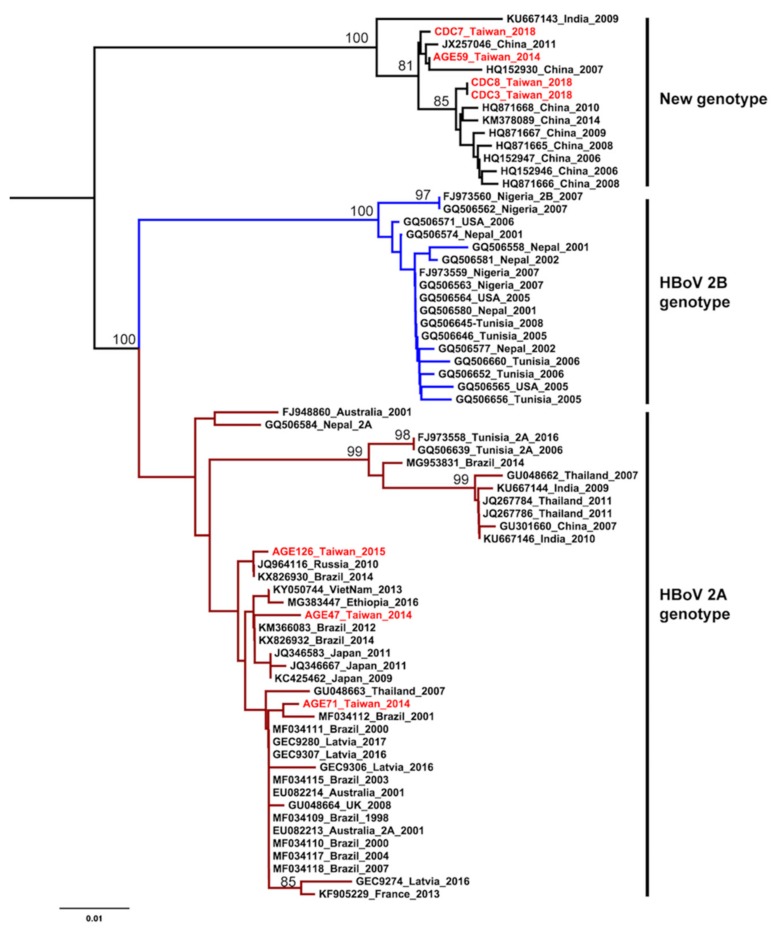
Phylogenetic analysis of human bocavirus 2 using the neighbor-joining method. The phylogenetic tree was reconstructed based on the HBoV2 partial VP1 gene. The stability of the tree topology was evaluated by using 1000 bootstrap replicates. Only bootstrap values greater than 75% are shown on the branch. The red texts represent HBoV2 isolates in Taiwan from the children with AGE. Brown and blue clades are represented HBoV2A and HBoV2B, respectively.

The HKY+G was estimated for the best-fit nucleotide substitution model to construct the phylogenetic trees and phylodynamic analysis. Both ML and NJ trees showed a consistent topology. The HBoV2 sequences from Taiwan have significant genetic variability and can be divided into two clusters. One cluster is together with genotype 2A from Brazil, Japan, Latvia, and Australia. The other one is genetically close to sequences from China and India, that form an independent cluster beyond the two known genotypes (Figure 1 and Figure 2). This new unclassified lineage includes sequences from Taiwan (CDC-2018 and AGE59-2014), China (e.g., AC: HQ152930 and HQ871668), and India (AC: KU667143). Furthermore, the mean nucleotide distance of the Taiwanese sequences in the independent cluster was 0.56% (0%–0.9%; Table 2). Nucleotide distances between that independent cluster and HBoV2A (W153; AC: EU082213) and HBoV2B (AC: FJ973560) was 6.65% and 9.05%, respective (Table 2). Based on the results of phylogenetic analysis and nucleotide distance, this independent cluster could be considered as a potential new HBoV2 genotype.

Before phylodynamic analysis, we used TempEst software (Available online: http://tree.bio.ed.ac.uk/) to investigate the temporal signal [24]. However, the root-to-tip regression analysis showed a negative correlation between the collection year and genetic divergence, suggesting that the dataset may not be appropriate for molecular clock analysis.

### 3.2. Selection Pressures in the HBoV2 VP1 Protein Genes

The selection pressures were estimated by the ratio of nonsynonymous substitutions (dN) to synonymous substitutions (dS). A dN/dS ratio of <1 represents negative selection; dN/dS ratio of 1 represents neutrality, and dN/dS ratio of >1 represents positive selection. No positive selection site was found in the dataset using SLAC, FEL methods with a *p*-value threshold of 0.05, and the FUBAR method with a posterior probability of 0.95. The dN/dS rate in the VP1 region was 0.080, suggesting that it was under purifying selection (Ka/Ks <1; Table 3).

## 4. Discussion

This is the first report showing that HBoV2 has been circulated in Taiwan. We identified the HBoV1 and HBoV2 DNA from stool samples of AGE children and investigated the genetic diversity and evolution of HBoV2 in Taiwan. Our results showed that the prevalence of HBoV in AGE children was 8.52%, whereas HBoV1 and HBoV2 were 6.25% and 2.27%, respectively. This is higher than the previous reports of AGE associated with HBoV in Taiwan (3.5%) [16], United States (3.4%) [25], and India (5.7%) [26].

In Taiwan, the dominant HBoV species associated with AGE is HBoV1, followed by HBoV2. Similar results have also been observed in the United States [25], India [26], Thailand [5], Latvia [9], and Bangladesh [27]. However, HBoV2 is the dominant species in some countries such as China [28], Nigeria and Tunisia [2], Australia [4], Russia [29], and Turkey [27]. Interestingly, the detection rate of HBoV2 is rather variable. A high detection rate has been observed in China, Nigeria, Tunisia, and Australia (17.2%–26%) [2,4,28], which is several times higher than that of Taiwan (2.27%). In general, HBoV1 and HBoV2 are the main species in pediatric gastrointestinal infections worldwide. However, HBoV3 and HBoV4 were undetectable in the present study, suggesting a possible low prevalence and frequency of these viruses in Taiwan. The real situation of HBoV3 and HBoV4 infection needs further study by increasing the number of samples and continuous surveillance.

HBoV2 can be further divided into 2A and 2B genotypes based on an average of 3.9% and 6.2% divergence for amino acids and nucleotides in the VP1/2 gene, respectively. [2]. Our phylogenetic analysis showed that the Taiwanese sequences have significant genetic variability. The Taiwan CDC sequences and AGE59 were phylogenetic close to the sequences from China and clustered into an independent lineage, while another part of the Taiwanese sequences belonged to HBoV2A genotype and clustered together with sequences from Brazil, Japan, Latvia, and Australia. This suggests multiple origins of HBoV2 in Taiwan. They were probably introduced into the country at different times, from different places.

According to the classification principle proposed by Kapoor et al., when HBoV strains show >8% protein and >10% nucleotide differences in the complete VP1 gene, they should be considered as a different species, whereas those showing >1.5% protein and >5% nucleotide differences should be considered as different genotypes [2]. Comparison of CDC 2018 and AGE59 sequences to HBoV2A (W153; AC: EU082213) and HBoV2B (AC: FJ973560) showed the nucleotide distances of 6.65% (6.4–6.8%) and 9.05% (8.9–9.1%), respectively (Table 2). Therefore, we consider that the independent cluster is probably a new genotype within HBoV2 and the new genotype tends to substitute the HBoV2A becoming a predominant genotype in Taiwan.

As described in previous literature, HBoVs are considered to be highly diverse and frequently recombinant pathogens [2,11]. However, we did not identify any positive selection site in this study using SLAC, FEL, and FUBAR methods. In addition, the dN/dS ratio in the VP1 region was 0.080, suggesting that the virus was under purifying selection (Ka/Ks <1) and the virus gene did not suffer strong evolutionary pressure.

## 5. Conclusions

This is the first report that reveals HBoV2 genetic diversity and phylogenetic relationships among AGE children in Taiwan. The HBoV2 strain may have been introduced into the country by multiple origins. We also propose a possible new HBoV2 genotype. Our results reinforce the understanding of HBoV2 molecular epidemiology and evolution.

## Figures and Tables

**Table 1:** Sequences list of the VP1 region of the human bocavirus 2 (HBoV2) used in this study.

Laboratory Numbers/Accession Numbers	Country	Source	Year
AGE-47, AGE-59, AGE-71, AGE-126	Taiwan	This study	2014
CDC3, CDC7, CDC8	Taiwan	Centers for Disease Control, Taiwan	2018
GEC-9274, GEC-9280, GEC-9306, GEC-9307	Latvia	Riga Stradins University	2016–2017
EU082213-4, FJ948860	Australia	NCBI	2001
FJ973558-60	Tunisia	NCBI	2016
FJ973559-60, GQ506562-3	Nigeria	NCBI	2007
GQ506584	Nigeria	NCBI	
GQ506564-5, GQ506571,	USA	NCBI	2005–2006
GQ506558, GQ506574, GQ506577, GQ506580-1	Nepal	NCBI	2001–2002
GQ506639, GQ506645-6, GQ506652, GQ506656, GQ506660	Tunisia	NCBI	2005–2008
GU048662-3, JQ267784, JQ267786	Thailand	NCBI	2007–2011
GU048664	UK	NCBI	2008
GU301660, HQ152930, HQ152946-7, HQ871665, HQ871666-8, JX257046, KM378089	China	NCBI	2006–2014
KC425462, JQ346583, JQ346667	Japan	NCBI	2009–2011
JQ964116	Russia	NCBI	2010
KF905229	France	NCBI	2013
KM366083, KX826930, KX826932, MF034109-12, MF034115, MF034117-8, MG953831	Brazil	NCBI	1998–2014
KU667143-4, KU667146	India	NCBI	2009–2010
KY050744	Viet Nam	NCBI	2013
MG383447	Ethiopia	NCBI	2016

**Table 2:** Divergence between the Taiwan HBoV2 sequences and genotypes for the VP1 gene region.

	HBoV2A^a^	HBoV2B^b^	AGE-47	AGE-71	AGE-126	AGE-59	CDC3	CDC7	CDC8
HBoV2A									
AGE-47	1.1%	6.4%		1.6%	1.1%	6.8%	7.3%	7.1%	7.3%
AGE-71	0.5%	6.2%	1.6%		1.4%	6.8%	7.3%	7.1%	7.3%
AGE-126	0.9%	5.7%	1.1%	1.4%		6.6%	7.1%	6.8%	7.1%
Novel HBoV2									
AGE-59	6.4%	9.1%	6.8%	6.8%	6.6%		0.9%	0.2%	0.9%
CDC3	6.6%	9.1%	7.3%	7.3%	7.1%	0.9%		0.7%	0%
CDC7	6.8%	8.9%	7.1%	7.1%	6.8%	0.2%	0.7%		0.7%
CDC8	6.8%	9.1%	7.3%	7.3%	7.1%	0.9%	0%	0.7%	

^a^ Human bocavirus 2 strain W153 (EU082213). ^b^ Human bocavirus 2b NI strain HBoV2B-NI-213 (FJ973560).

**Table 3:** Selection sites detected in the VP1 protein region of HBoV2.

Positively Selected Sites			No. of NEGATIVELY Selected Sites			Mean dN/dS	
SLAC^a^	FEL^a^	FUBA^b^	SLAC^a^	FEL^a^	FUBA^b^		
Non	Non	Non	16	34	26		0.080

^a^*p*-value of <0.05. ^b^ Posterior probability of ≥0.95.
